# Supplementary figures and images for: Effects of PAHs on meiofauna from three estuaries with different levels of urbanization in the South Atlantic
Source: PeerJ. 2022 Dec 2;10:e14407. doi: 10.7717/peerj.14407 (PMC9744168; doi:10.7717/peerj.14407)

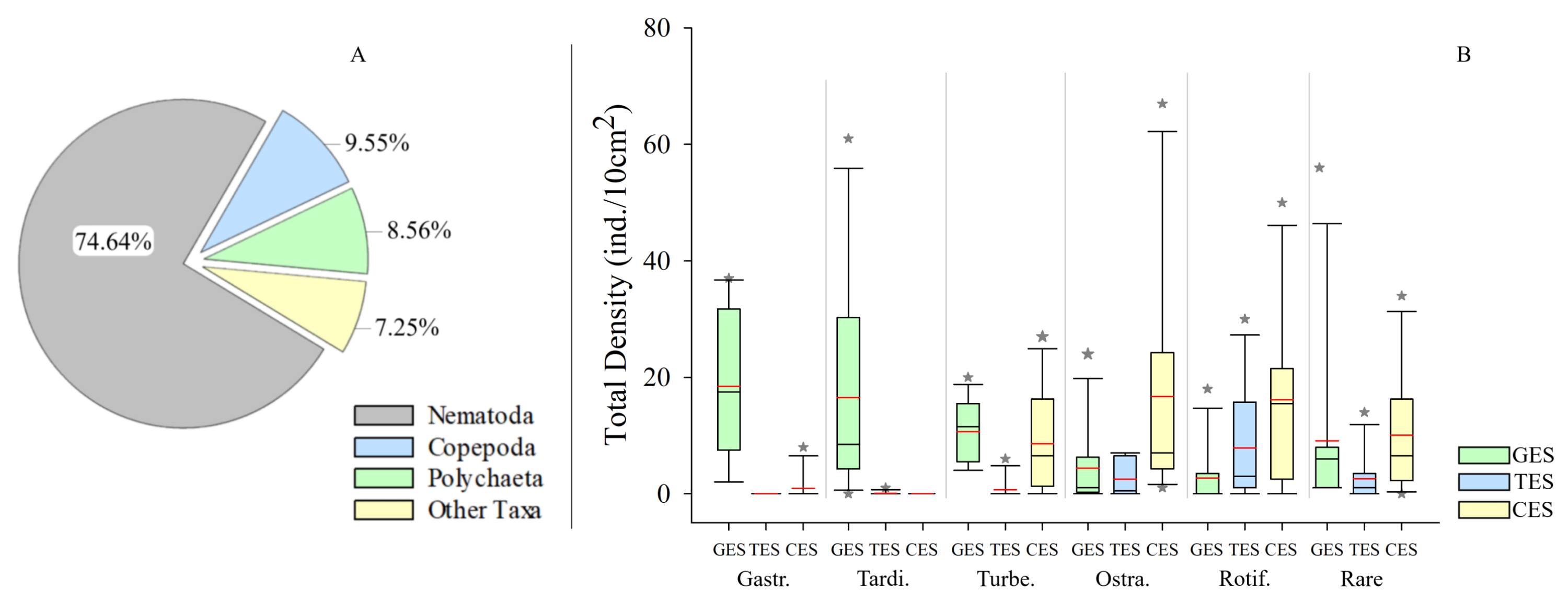

Supplement: Supplemental Information 1 — Density of groups recorded in each estuary. (A) Pie chart indicating the relative group abundances of all estuaries. (B) Whisker boxes represent upper/lower quartiles with detailed focus on Other Taxa (<2% of the total density) from panel A, Median (solid black line), Average (in red). Vertical lines extending from each box represent the minimum and maximum value, “*” are outliers that outranged the box limits. GES, Goiana estuarine system; TES, Timbó estuarine system; CES, Capibaribe estuarine system; Gastr., Gastrotricha; Tardi., Tardigrada; Turbe., Turbellaria; Ostra., Ostracoda; Rotif., Rotifera. [file peerj-10-14407-s001.png]

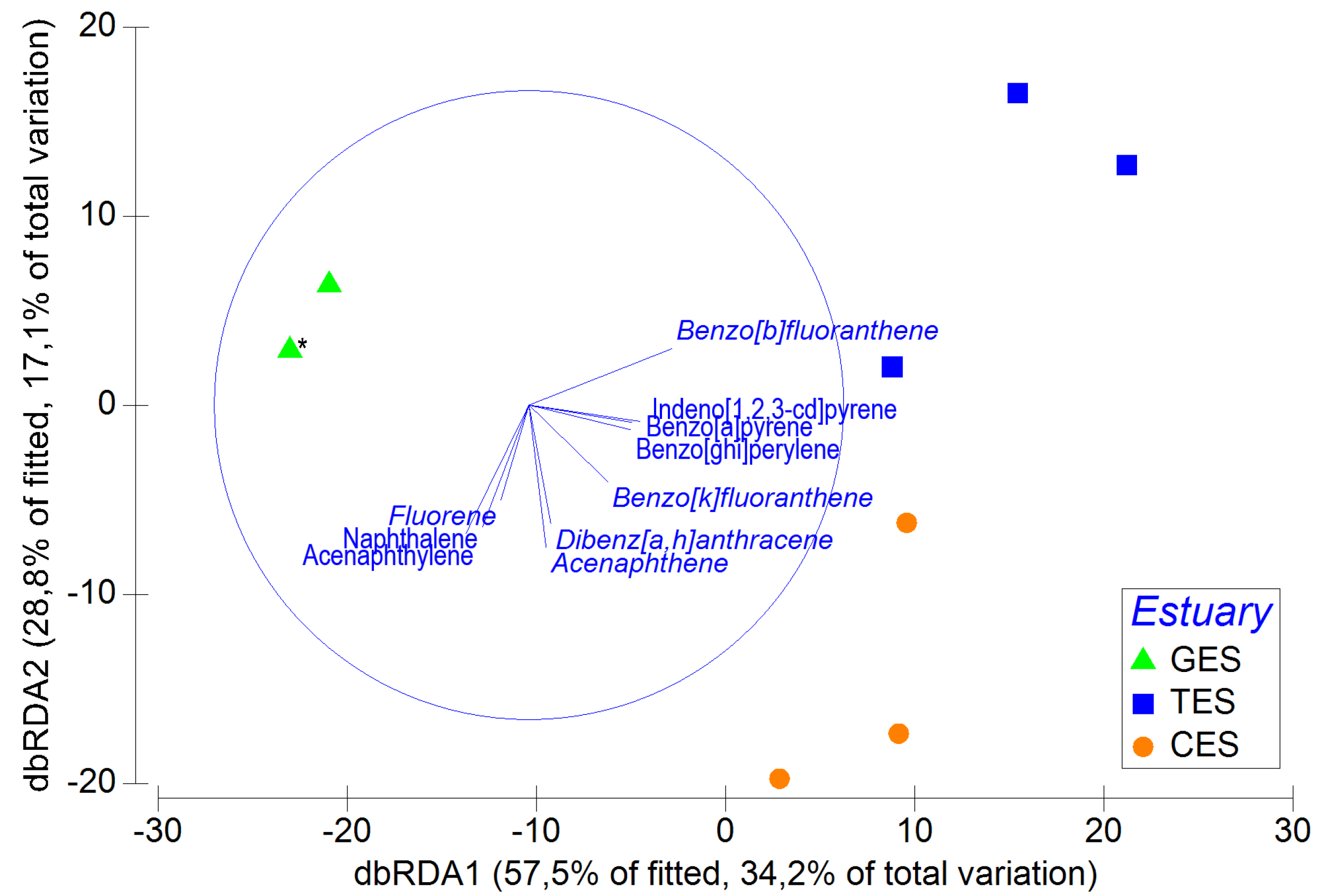

Supplement: Supplemental Information 2 — Distance-based redundancy analysis (dbRDA), representing DISTLM model based on the meiofauna assemblage data and fitted PAHs with their vector (strength and direction of effect of the variable on the ordination plot). The overlap of factors was restricted in order to include variables that have a correlation greater than 0.3. GES, Goiana estuarine system; TES, Timbó estuarine system; CES, Capibaribe estuarine system. *Two points are overlapping on GES as they do not present PAHs. [file peerj-10-14407-s002.png]

Summary Figure:

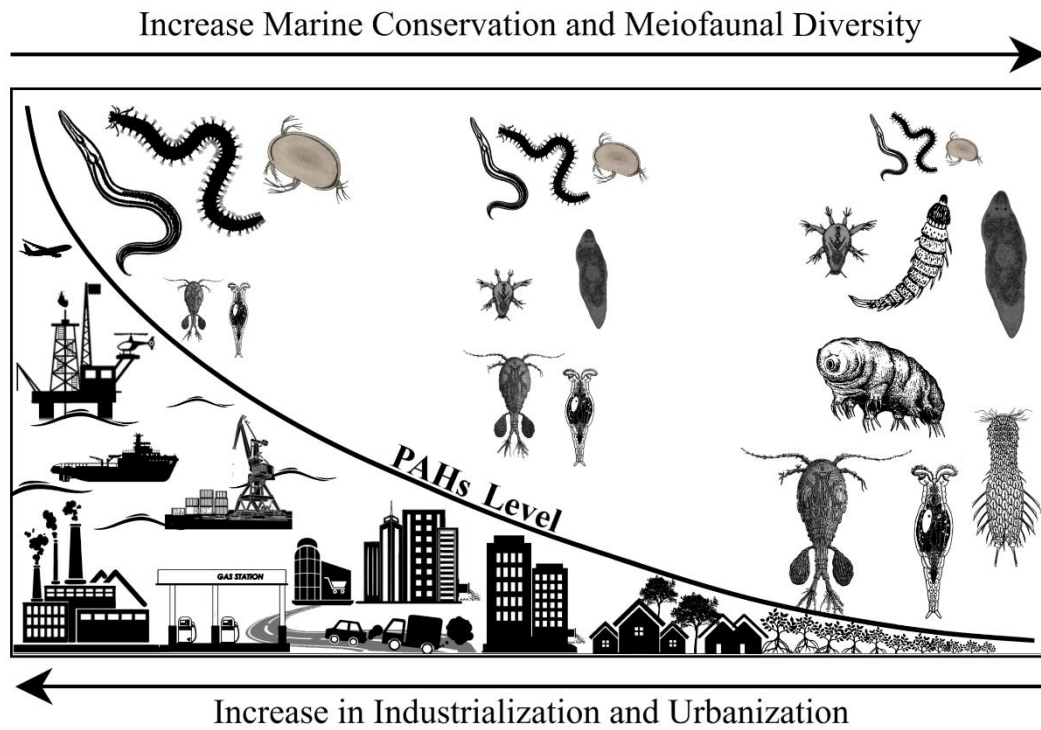

Supplement: Supplemental Information 14 [file peerj-10-14407-s014.pdf]
