# Supplementary material for: Effects of PAHs on meiofauna from three estuaries with different levels of urbanization in the South Atlantic
Source: PeerJ. 2022 Dec 2;10:e14407. doi: 10.7717/peerj.14407 (PMC9744168; doi:10.7717/peerj.14407)
Supplement: Supplemental Information 3 — Average values (±SE) of the environmental variables of the three estuaries. Values of P (perm) < 0.05 are in bold. The letters represent groups of significant differences between the studied areas. GES, Goiana estuarine system; TES, Timbó estuarine system; CES, Capibaribe estuarine system. $\rm \sum PAH$∑PAH, sum of polycyclic aromatic hydrocarbons; DO, dissolved oxygen; OM, organic matter; Temp, temperature. [file peerj-10-14407-s003.docx]

| Area/Station | ∑PAH | DO | pH | OM | Temp. | Salinity |
| --- | --- | --- | --- | --- | --- | --- |
|  | ng/g^-1^ | mg/L |  | mg/g | Cº |  |
| GES | 0.55 ± 0.67 ^a^ | 4.45 ± 0.44 ^a^ | 8.40 ± 0.04 ^a^ | 1.87 ± 0.35 ^a^ | 29.13 ± 0.08 | 27.33 ± 2.16 |
| TES | 139.17 ± 71.43 ^b^ | 5.43 ± 0.49 ^a^ | 8.39 ± 0.06 ^a^ | 6.88 ± 1.12 ^b^ | 29.03 ± 0.04 | 30.67 ± 3.56 |
| CES | 674.81 ± 331.31 ^b^ | 17.67 ± 2.04 ^b^ | 5.81 ± 0.02 ^b^ | 11.77 ± 3.04 ^b^ | 29.47 ± 0.27 | 27.57 ± 3.78 |
| *p* | **0.01** | **0.024** | **0.039** | **0.014** | 0.103 | 0.349 |
| *Pseudo-F* | 21.712 | 63.793 | 4146.9 | 21.609 | 3.381 | 1.364 |
| *PermDisp* | 0.9214 | 0.3889 | 0.071 | 0.7611 | 0.0245 | 0.7779 |
