# Supplementary material for: Effects of PAHs on meiofauna from three estuaries with different levels of urbanization in the South Atlantic
Source: PeerJ. 2022 Dec 2;10:e14407. doi: 10.7717/peerj.14407 (PMC9744168; doi:10.7717/peerj.14407)
Supplement: Supplemental Information 4 — Average values (±SE) of the granulometric fractions in the three estuaries. Result of PERMANOVA, PERMIDISP on the granulometric matrix of estuaries. Values of P(perm) < 0.05 are in bold. The small cap (a and b) letters represent groups of significant differences between the study areas and each granulometric size. GES, Goiana estuarine system; TES, Timbó estuarine system; CES, Capibaribe estuarine system; VCS, very coarse sand; CS, coarse sand; MS, medium sand; FS, fine sand; VFS, very fine sand. [file peerj-10-14407-s004.docx]

| Estuary | Gravel | Sand | VCS | CS | MS | FS | VFS | Silt-clay |
| --- | --- | --- | --- | --- | --- | --- | --- | --- |
|  | % | | | | | | | |
| GES | 2 | 96 | 2.13 | 16.03 | 43.23 | 33.96 | 0.67 | 1.97 |
|  | ± 2.27 | ± 3.28 ^a^ | ± 1.45 | ± 9.62 | ± 12.5 | ± 24.61 | ± 0.11^a^ | ± 1.0 ^a^ |
| TES | 5.13 | 83.9 | 5.8 | 13.77 | 19.7 | 27.83 | 16.87 | 10.97 |
|  | ± 4.7 | ± 3.4 ^b^ | ± 3.01 | ± 5.98 | ±5.27 | ± 7.27 | ± 8.72 ^b^ | ± 4.52 ^ab^ |
| CES | 9.17 | 64 | 5.9 | 13.67 | 17.93 | 15.1 | 11.5 | 26.73 |
|  | ± 6.63 | ± 14.99 ^b^ | ± 3.48 | ± 6.88 | ± 7.48 | ± 2.18 | ± 4.67 ^b^ | ± 10.7 ^b^ |
| *p* | 0.384 | **0.02** | 0.459 | 0.956 | 0.258 | 0.482 | **0.043** | **0.048** |
| *Pseudo-F* | 1.1259 | 5.2086 | 1.1466 | 5.74E-02 | 2.913 | 0.62938 | 7.8821 | 9.3009 |
| *PermDisp* | 0.8707 | 0.2695 | 0.8873 | 0.1701 | 0.7261 | 0.1298 | 0.0522 | 0.4192 |
