# Supplementary material for: Effects of PAHs on meiofauna from three estuaries with different levels of urbanization in the South Atlantic
Source: PeerJ. 2022 Dec 2;10:e14407. doi: 10.7717/peerj.14407 (PMC9744168; doi:10.7717/peerj.14407)
Supplement: Supplemental Information 5 — PERMANOVA and PAIR-WISE results for the ecological indices in the study estuaries. The analysis factor was area (Estuary). Values of P(perm) < 0.05 are in bold. GES, Goiana estuarine system; TES, Timbó estuarine system; CES, Capibaribe estuarine system; df, degrees of freedom; MS, mean squares; Res, residual. [file peerj-10-14407-s005.docx]

| Estuary | Med. (±SE) Estuary | Station | Med. (±SE) Station | EcoQ |
| --- | --- | --- | --- | --- |
|  |  | St1 | 9 ± 0.54 | Moderate |
| GES | 8.75 ± 0.5 | St2 | 9 ± 0.2 | Moderate |
|  |  | St3 | 8.25 ± 0.52 | Moderate |
|  |  | St1 | 6.25 ± 0.69 | Poor |
| TES | 5.33 ± 0.55 | St2 | 4.75 ± 0.24 | Poor |
|  |  | St3 | 5 ± 0.35 | Poor |
|  |  | St1 | 8.5 ± 0.14 | Moderate |
| CES | 7.83 ± 0.46 | St2 | 6.25 ± 0.38 | poor |
|  |  | St3 | 8.75 ± 0.24 | Moderate |
