# Supplementary material for: Effects of PAHs on meiofauna from three estuaries with different levels of urbanization in the South Atlantic
Source: PeerJ. 2022 Dec 2;10:e14407. doi: 10.7717/peerj.14407 (PMC9744168; doi:10.7717/peerj.14407)
Supplement: Supplemental Information 6 — Mean values (±SE) of richness within each estuary and in their respective stations, in addition to the ecological quality corresponding to the richness of each station. GES, Goiana estuarine system; TES, Timbó estuarine system; CES, Capibaribe estuarine system; Med, average; EcoQ, environmental quality status; St, Station. [file peerj-10-14407-s006.docx]

|  |  | PERMANOVA | | | |  | PAIR-WISE | |
| --- | --- | --- | --- | --- | --- | --- | --- | --- |
| Ecological index | Source | df | MS | pseudo-F | P (perm) | Groups | t | P (perm) |
| Richness - S | Estuary | 2 | 143.02 | 14.31 | **0.0002** | GES, TES | 4.4379 | **0.0007** |
|  | Res. | 33 | 9.995 |  |  | GES, CES | 1.4402 | 0.1621 |
|  | Total | 35 |  |  |  | TES, CES | 3.7744 | **0.0018** |
| Density - N | Estuary | 2 | 850.14 | 9.7861 | **0.0002** | GES, TES | 1.6754 | 0.1060 |
|  | Res. | 33 | 86.872 |  |  | GES, CES | 3.1014 | **0.0065** |
|  | Total | 35 |  |  |  | TES, CES | 4.2221 | **0.0002** |
| Shannon ’ H' | Estuary | 2 | 118.86 | 8.2434 | **0.0024** | GES, TES | 3.3466 | **0.0053** |
|  | Res. | 33 | 14.418 |  |  | GES, CES | 3.4752 | **0.0040** |
|  | Total | 35 |  |  |  | TES, CES | 0.43717 | 0.6684 |
| Evenness ’ J' | Estuary | 2 | 81.323 | 6.264 | **0.0061** | GES, TES | 1.0207 | 0.3174 |
|  | Res. | 33 | 12.983 |  |  | GES, CES | 3.0416 | **0.0082** |
|  | Total | 35 |  |  |  | TES, CES | 2.7726 | **0.0130** |
