# Supplementary material for: Effects of PAHs on meiofauna from three estuaries with different levels of urbanization in the South Atlantic
Source: PeerJ. 2022 Dec 2;10:e14407. doi: 10.7717/peerj.14407 (PMC9744168; doi:10.7717/peerj.14407)
Supplement: Supplemental Information 7 — Group of environmental variables, selected by the DistLM-BEST analysis, that most correlate with the estuarine fauna. The BEST procedure was used on similarity matrices based on meiofauna density. RSS, Residual Sum of Squares; No. Vars, number of variables; $\rm \sum PAH$∑PAHs, sum of polycyclic aromatic hydrocarbons; DO, dissolved oxygen; VCSand, very coarse sand; OM, organic matter; CSand, coarse sand; MSand, medium sand. [file peerj-10-14407-s007.docx]

| **R^2^** | **RSS** | **No. Vars** | **Variable Selection** |
| --- | --- | --- | --- |
| 0.26226 | 21170 | 1 | ∑PAHs |
| 0.39501 | 17360 | 2 | ∑PAHs; pH |
| 0.47378 | 15100 | 3 | DO; pH; VCSand |
| 0.52768 | 13553 | 4 | DO; pH; VCSand; Silt-clay |
| 0.56147 | 12584 | 5 | DO; pH; OM; VCSand; Silt-clay |
| 0.59516 | 11617 | 6 | DO; pH; OM; Sand; VCSand; Silt-clay |
| **0.60939** | 11209 | 7 | DO; pH; Sand; VCSand; Csand; MSand; Silt-clay |
