# Supplementary material for: Effects of PAHs on meiofauna from three estuaries with different levels of urbanization in the South Atlantic
Source: PeerJ. 2022 Dec 2;10:e14407. doi: 10.7717/peerj.14407 (PMC9744168; doi:10.7717/peerj.14407)
Supplement: Supplemental Information 8 — Spearman rank of correlation values between environmental data, and each meiofaunal taxa. Nemat., Nematoda; Copep., Copepoda; Rotif., Rotifera; Turbe., Turbellaria; Tardi., Tardigrada; Gastr., Gastrotricha; Ostra., Ostracoda; Halac., Halacaroidea; Naupl., Nauplius; Oligo., Oligochaeta; Cnida., Cnidaria; Polyc., Polychaeta; Amphi., Amphipoda; Sipun., Sipuncula; Kinor., Kinorhyncha; Priapu., Priapulida; Total PAHs, total polycyclic aromatic hydrocarbons; DO, dissolved oxygen; OM, organic matter; Temp., temperature; VCSand, very coarse sand; CSand, coarse sand; MSand, medium sand; FSand, fine sand; VFSand., very fine sand. Significant values are represented by: *p < 0.05, **p < 0.01, ***p < 0.001. [file peerj-10-14407-s008.docx]

**Supplementary Table A1**. Spearman rank of correlation values between environmental data, and each meiofaunal taxa. Nemat., Nematoda; Copep., Copepoda; Rotif., Rotifera; Turbe., Turbellaria; Tardi., Tardigrada; Gastr., Gastrotricha; Ostra., Ostracoda; Halac., Halacaroidea; Naupl., Nauplius; Oligo., Oligochaeta; Cnida., Cnidaria; Polyc., Polychaeta; Amphi., Amphipoda; Sipun., Sipuncula; Kinor., Kinorhyncha; Priapu., Priapulida; Total PAHs, Total Polycyclic Aromatic Hydrocarbons; DO, dissolved oxygen; OM, organic matter; Temp., Temperature; VCSand, Very Coarse Sand; CSand, Coarse Sand; MSand, Medium Sand; FSand, Fine Sand; VFSand., Very Fine Sand. Significant values are represented by: *p<0.05, **p<0.01, ***p<0.001.

|  | Nemat. | Copep. | Rotif. | Turbe. | Tardi. | Gastr. | Ostra. | Halac. | Naupl. | Oligo. | Cnida. | Polyc. | Amphi. | Sipun. | Kinor. | Priapu. |
| --- | --- | --- | --- | --- | --- | --- | --- | --- | --- | --- | --- | --- | --- | --- | --- | --- |
| Total PAHs | 0,52** | -0,31 | 0,58*** | -0,09 | -0,73*** | -0,67*** | 0,47** | -0,46** | 0,02 | 0,32 | 0,58*** | 0,73*** | 0,07 | -0,43** | -0,23 | -0,23 |
| Salinity | 0,03 | -0,07 | 0,09 | -0,37* | 0,004 | -0,13 | -0,20 | -0,15 | -0,03 | -0,02 | 0,18 | -0,02 | 0,26 | -0,06 | 0,00 | -0,20 |
| DO | 0,38* | -0,21 | 0,48** | -0,15 | -0,67*** | -0,57*** | 0,32 | -0,52** | -0,11 | 0,23 | 0,53*** | 0,66*** | -0,13 | -0,31 | -0,07 | -0,26 |
| pH | -0,38* | 0,17 | -0,27 | -0,16 | 0,49** | 0,34* | -0,48** | 0,28 | -0,08 | -0,20 | -0,36* | -0,53*** | 0,26 | 0,26 | 0,20 | -0,03 |
| OM | 0,29 | -0,33* | 0,35* | -0,19 | -0,77*** | -0,71*** | 0,31 | -0,46** | -0,13 | 0,23 | 0,26 | 0,58*** | 0,07 | -0,41* | -0,20 | -0,13 |
| Temp. | 0,45** | -0,10 | 0,11 | 0,42* | -0,18 | -0,02 | 0,39* | -0,09 | 0,16 | 0,41* | 0,28 | 0,43** | -0,20 | 0,01 | 0,07 | 0,07 |
| Gravel | 0,18 | -0,26 | 0,29 | -0,02 | -0,60*** | -0,53*** | 0,34* | -0,39* | 0,03 | 0,16 | 0,21 | 0,49** | -0,07 | -0,34* | -0,26 | 0,07 |
| Sand | -0,46** | 0,24 | -0,59*** | 0,19 | 0,75*** | 0,76*** | -0,41* | 0,48** | -0,12 | -0,34* | -0,60*** | -0,61*** | -0,13 | 0,44** | 0,26 | 0,13 |
| VCSand | -0,14 | -0,43** | 0,01 | -0,28 | -0,52** | -0,50** | 0,02 | -0,38* | -0,26 | -0,07 | -0,21 | 0,32 | 0,13 | -0,34* | -0,26 | 0,00 |
| CSand | -0,16 | -0,15 | -0,35* | -0,16 | -0,04 | -0,02 | -0,06 | -0,09 | -0,01 | -0,23 | -0,22 | -0,12 | -0,13 | -0,10 | -0,26 | 0,26 |
| MSand | -0,25 | 0,01 | -0,49** | 0,19 | 0,54*** | 0,57*** | -0,18 | 0,28 | 0,02 | -0,27 | -0,39* | -0,32 | -0,13 | 0,23 | 0,00 | 0,20 |
| FSand | -0,23 | 0,16 | -0,04 | -0,24 | 0,23 | 0,15 | -0,37* | 0,10 | -0,24 | -0,16 | -0,13 | -0,27 | 0,13 | 0,17 | 0,26 | -0,26 |
| VFSand | 0,21 | -0,16 | 0,35* | -0,54* | -0,71*** | -0,78*** | 0,05 | -0,47** | -0,13 | 0,16 | 0,34* | 0,30 | 0,20 | -0,37* | -0,13 | -0,20 |
| Silt | 0,50* | -0,19 | 0,54*** | -0,21 | -0,75*** | -0,76*** | 0,38* | -0,46** | 0,11 | 0,39* | 0,60*** | 0,55*** | 0,13 | -0,41* | -0,20 | -0,13 |
