# Supplementary material for: Effects of PAHs on meiofauna from three estuaries with different levels of urbanization in the South Atlantic
Source: PeerJ. 2022 Dec 2;10:e14407. doi: 10.7717/peerj.14407 (PMC9744168; doi:10.7717/peerj.14407)
Supplement: Supplemental Information 9 — Spearman correlation values between each individual concentration of PAHs, and each meiofaunal taxa. Nemat., Nematoda; Copep., Copepoda; Rotif., Rotifera; Turbe., Turbellaria; Tardi., Tardigrada; Gastr., Gastrotricha; Ostra., Ostracoda; Halac., Halacaroidea; Naupl., Nauplius; Oligo., Oligochaeta; Cnida., Cnidaria; Polyc., Polychaeta; Amphi., Amphipoda; Sipun., Sipuncula; Kinor., Kinorhyncha; Priapu., Priapulida; DO, dissolved oxygen; OM, organic matter; Temp., temperature; VCSand, very coarse sand; CSand, coarse sand; MSand, medium sand; FSand, fine sand; VFSand., very fine sand. Significant values are represented by: *p < 0.05, **p < 0.01, ***p < 0.001. [file peerj-10-14407-s009.docx]

**Supplementary Table A2**. Spearman correlation values between each individual concentration of PAHs, and each meiofaunal taxa. Nemat., Nematoda; Copep., Copepoda; Rotif., Rotifera; Turbe., Turbellaria; Tardi., Tardigrada; Gastr., Gastrotricha; Ostra., Ostracoda; Halac., Halacaroidea; Naupl., Nauplius; Oligo., Oligochaeta; Cnida., Cnidaria; Polyc., Polychaeta; Amphi., Amphipoda; Sipun., Sipuncula; Kinor., Kinorhyncha; Priapu., Priapulida; DO, dissolved oxygen; OM, organic matter; Temp., Temperature; VCSand, Very Coarse Sand; CSand, Coarse Sand; MSand, Medium Sand; FSand, Fine Sand; VFSand., Very Fine Sand. Significant values are represented by: *p<0.05, **p<0.01, ***p<0.001.

|  | Nemat. | Copep. | Rotif. | Turbe. | Tardi. | Gastr. | Ostra. | Halac. | Naupl. | Oligo. | Cnida. | Polyc. | Amphi. | Sipun. | Kinor. | Priapu. |
| --- | --- | --- | --- | --- | --- | --- | --- | --- | --- | --- | --- | --- | --- | --- | --- | --- |
| 2-Methyl Naphthalene | 0,55*** | -0,30 | 0,56*** | -0,01 | -0,63*** | -0,57*** | 0,51** | -0,37* | 0,06 | 0,32 | 0,58*** | 0,71*** | 0,07 | -0,40* | -0,23 | -0,23 |
| Acenaphthylene | 0,61*** | -0,28 | 0,51** | 0,00 | -0,64*** | -0,58*** | 0,49** | -0,39* | 0,11 | 0,43** | 0,61*** | 0,69*** | 0,07 | -0,36* | -0,17 | -0,17 |
| Acenaphthene | 0,56*** | -0,28 | 0,41* | 0,27 | -0,48** | -0,33 | 0,55*** | -0,30 | 0,10 | 0,39* | 0,49** | 0,73*** | -0,12 | -0,24 | -0,12 | -0,12 |
| Fluorene | 0,56*** | -0,24 | 0,51** | 0,05 | -0,58*** | -0,50** | 0,52** | -0,31 | 0,07 | 0,31 | 0,57*** | 0,66*** | 0,00 | -0,35* | -0,20 | -0,20 |
| Phenanthrene | 0,52** | -0,31 | 0,58*** | -0,09 | -0,73*** | -0,67*** | 0,47** | -0,46** | 0,02 | 0,32 | 0,58*** | 0,73*** | 0,07 | -0,43** | -0,23 | -0,23 |
| Anthracene | 0,55*** | -0,34* | 0,44** | 0,01 | -0,64*** | -0,56*** | 0,46** | -0,39* | 0,04 | 0,39* | 0,49** | 0,70*** | 0,07 | -0,36* | -0,17 | -0,17 |
| Fluoranthene | 0,53*** | -0,30 | 0,55*** | -0,11 | -0,75*** | -0,70*** | 0,45** | -0,49** | 0,03 | 0,37* | 0,59*** | 0,72*** | 0,07 | -0,42* | -0,20 | -0,20 |
| Pyrene | 0,53*** | -0,30 | 0,55*** | -0,11 | -0,75*** | -0,70*** | 0,45** | -0,49** | 0,03 | 0,37* | 0,59*** | 0,72*** | 0,07 | -0,42* | -0,20 | -0,20 |
| Benzo[a]anthracene | 0,53*** | -0,27 | 0,58*** | -0,14 | -0,74*** | -0,72*** | 0,43** | -0,47** | 0,07 | 0,39* | 0,61*** | 0,67*** | 0,13 | -0,42* | -0,20 | -0,20 |
| Chrysene | 0,53*** | -0,30 | 0,55*** | -0,11 | -0,75*** | -0,70*** | 0,45** | -0,49** | 0,03 | 0,37* | 0,59*** | 0,72*** | 0,07 | -0,42* | -0,20 | -0,20 |
| Benzo[b]fluoranthene | 0,53*** | -0,30 | 0,55*** | -0,11 | -0,75*** | -0,70*** | 0,45** | -0,49** | 0,03 | 0,37* | 0,59*** | 0,72*** | 0,07 | -0,42* | -0,20 | -0,20 |
| Benzo[k]fluoranthene | 0,53*** | -0,30 | 0,55*** | -0,11 | -0,75*** | -0,70*** | 0,45** | -0,49** | 0,03 | 0,37* | 0,59*** | 0,72*** | 0,07 | -0,42* | -0,20 | -0,20 |
| Benzo[a]pyrene | 0,53*** | -0,30 | 0,55*** | -0,11 | -0,75*** | -0,70*** | 0,45** | -0,49** | 0,03 | 0,37* | 0,59*** | 0,72*** | 0,07 | -0,42* | -0,20 | -0,20 |
| Indeno[1,2,3-cd]pyrene | 0,53*** | -0,30 | 0,55*** | -0,11 | -0,75*** | -0,70*** | 0,45** | -0,49** | 0,03 | 0,37* | 0,59*** | 0,72*** | 0,07 | -0,42* | -0,20 | -0,20 |
| Dibenz[a,h]anthracene | 0,53*** | -0,30 | 0,55*** | -0,11 | -0,75*** | -0,70*** | 0,45** | -0,49** | 0,03 | 0,37* | 0,59*** | 0,72*** | 0,07 | -0,42* | -0,20 | -0,20 |
| Benzo[ghi]perylene | 0,53*** | -0,30 | 0,55*** | -0,11 | -0,75*** | -0,70*** | 0,45** | -0,49** | 0,03 | 0,37* | 0,59*** | 0,72*** | 0,07 | -0,42* | -0,20 | -0,20 |
| Naphthalene | 0,55*** | -0,34 | 0,44** | 0,01 | -0,64*** | -0,56*** | 0,46** | -0,39* | 0,04 | 0,39* | 0,49** | 0,70*** | 0,07 | -0,36* | -0,17 | -0,17 |
