# Supplementary material for: Effects of PAHs on meiofauna from three estuaries with different levels of urbanization in the South Atlantic
Source: PeerJ. 2022 Dec 2;10:e14407. doi: 10.7717/peerj.14407 (PMC9744168; doi:10.7717/peerj.14407)
Supplement: Supplemental Information 10 — Group of PAHs, selected by the DistLM analysis, which most correlated with estuarine fauna. The BEST procedure was used on similarity matrices based on meiofauna density. RSS, Residual Sum of Squares; No. Vars, number of variables; BbF, Benzo[b]fluoranthene; A, Anthracene; DA, Dibenz[a,h]anthracene; ghi, Benzo[ghi]perylene; BaA, Benzo[a]anthracene; BkF, Benzo[k]fluoranthene; IP, Indeno[1,2,3-cd]pyrene; AY, Acenaphthylene; F, Fluorene. [file peerj-10-14407-s010.docx]

| **R^2^** | **RSS** | **No. Vars** | **Variable Selection** |
| --- | --- | --- | --- |
| 0.24024 | 21801 | 1 | BbF |
| 0.46448 | 15367 | 2 | A; BbF |
| 0.50893 | 14091 | 3 | A; BbF; DA |
| 0.54333 | 13104 | 4 | A; BbF; DA; ghi |
| 0.57024 | 12332 | 5 | A; BaA; BkF; IP; DA |
| **0.59441** | 11639 | 6 | AY; F; A; BbF; DA; ghi |
