# Supplementary material for: Effects of PAHs on meiofauna from three estuaries with different levels of urbanization in the South Atlantic
Source: PeerJ. 2022 Dec 2;10:e14407. doi: 10.7717/peerj.14407 (PMC9744168; doi:10.7717/peerj.14407)
Supplement: Supplemental Information 11 — Spearman correlation values between environmental data and diversity indices registered in estuaries: Density (ind./10 cm2), Shannon Index (H), meiofauna richness (S) and group equitability (J). Significant values are represented by: *p < 0.05, **p < 0.01, ***p < 0.001. [file peerj-10-14407-s011.docx]

|  | Density | Shannon | Richness | Equitability |
| --- | --- | --- | --- | --- |
|  | (ind./10cm^2^) | (H) | (S) | (J) |
| ∑ PAH | **0.450**** | -0.161 | -0.111 | **-0.422*** |
| Salinity | 0.004 | -0.095 | -0.075 | -0.067 |
| DO | **0.354*** | -0.194 | -0.141 | **-0.407*** |
| pH | -0.196 | 0.036 | -0.011 | 0.271 |
| OM | 0.214 | **-0.349*** | -0.32 | **-0.491**** |
| Temp. | **0.457*** | 0.228 | 0.258 | -0.224 |
| Gravel | 0.108 | -0.188 | -0.185 | -0.063 |
| Sand | **-0.389** | 0.185 | 0.148 | 0.316 |
| VCSand | **-0.237** | **-0.450**** | **-0.468**** | -0.138 |
| CSand | -0.265 | -0.208 | -0.182 | -0.05 |
| MSand | -0.27 | 0.174 | 0.163 | 0.315 |
| FSand | -0.157 | -0.064 | -0.079 | -0.021 |
| VFSand | -0.148 | **-0.456**** | **-0.409*** | **-0.608***** |
| Silt-clay | **0.437**** | -0.205 | -0.155 | **-0.489**** |
