# Supplementary material for: Effects of PAHs on meiofauna from three estuaries with different levels of urbanization in the South Atlantic
Source: PeerJ. 2022 Dec 2;10:e14407. doi: 10.7717/peerj.14407 (PMC9744168; doi:10.7717/peerj.14407)
Supplement: Supplemental Information 12 — Spearman correlation values between environmental data and diversity indices registered in estuaries: Density (ind./10 cm2), Shannon Index (H), meiofauna richness (S) and group equitability (J). Significant values are represented by: *p < 0.05, **p < 0.01, ***p < 0.001. [file peerj-10-14407-s012.docx]

**Supplementary Table A3**. Spearman correlation values between environmental data and diversity indices registered in estuaries: Density (ind./10cm^2^), Shannon Index (H), meiofauna richness (S) and group equitability (J). Significant values are represented by: *p<0.05, **p<0.01, ***p<0.001.

|  | Density | Shannon | Richness | Equitability |
| --- | --- | --- | --- | --- |
|  | (N) | Index (H) | (S) | (J) |
| 2-Methyl Naphthalene | -0,08** | 0,49 | -0,03 | -0,43** |
| Acenaphthylene | -0,06*** | 0,55 | -0,01 | -0,51** |
| Acenaphthene | 0,10** | 0,52 | 0,14 | -0,29 |
| Fluorene | -0,05** | 0,51 | 0,01 | -0,43** |
| Phenanthrene | -0,16** | 0,45 | -0,11 | -0,41* |
| Anthracene | -0,10** | 0,49 | -0,05 | -0,48** |
| Fluoranthene | -0,17** | 0,46 | -0,12 | -0,44** |
| Pyrene | -0,17** | 0,46 | -0,12 | -0,44** |
| Benzo[a]anthracene | -0,16** | 0,47 | -0,12 | -0,46** |
| Chrysene | -0,17** | 0,46 | -0,12 | -0,44** |
| Benzo[b]fluoranthene | -0,17** | 0,46 | -0,12 | -0,44** |
| Benzo[k]fluoranthene | -0,17** | 0,46 | -0,12 | -0,44** |
| Benzo[a]pyrene | -0,17** | 0,46 | -0,12 | -0,44** |
| Indeno[1,2,3-cd]pyrene | -0,17** | 0,46 | -0,12 | -0,44** |
| Dibenz[a,h]anthracene | -0,17** | 0,46 | -0,12 | -0,44** |
| Benzo[ghi]perylene | -0,17** | 0,46 | -0,12 | -0,44** |
| Naphthalene | -0,10** | 0,49 | -0,05 | -0,48** |
